# Efficacy of field-based surveys for detecting and assessing demographics of giant snakes (*Malayopython reticulatus*) in Malaysia

**DOI:** 10.1038/s41598-026-57269-9

**Published:** 2026-06-08

**Authors:** Richard Burger, Daniel J. D. Natusch, Richard Shine, Ian P. Vaughan, Symphorosa Sipangkui, Benoit Goossens

**Affiliations:** 1 Organisms and Environment Division, Cardiff School of Biosciences, Cardiff University, Sir Martin Evans Building, Museum Avenue, Cardiff, CF10 3AX UK; 2 Danau Girang Field Centre, c/o Sabah Wildlife Department, Wisma MUIS, Block B 5th Floor, Kota Kinabalu, 88100 Sabah Malaysia; 3 IUCN SSC Snake Specialist Group, 1/26 Florence St, Cairns City, 4870 QLD Australia; 4 School of Natural Sciences, Macquarie University, Sydney, 2109 NSW Australia; 5 Sabah Wildlife Department, Kota Kinabalu, Sabah Malaysia

**Keywords:** CITES, Malaysia, Non-detriment finding, Oil palm plantation, *Malayopython reticulatus*, Reptile, Snake, Sustainable harvest, Wildlife trade, Ecology, Ecology, Zoology

## Abstract

Evaluating sustainable levels of offtake is central to regulating the commercial harvest of wildlife species, but methods developed for temperate-zone mammal and bird species may be inapplicable to tropical rainforest reptiles. For example, population viability models require information on abundance and on rates of growth, maturation, survival, and reproduction. For some intensively harvested reptiles - especially well-camouflaged ambush predators in densely vegetated habitats - such data are difficult to obtain. The feasibility of gathering this information is central to current debates about how best to evaluate the sustainability of commercial harvesting of reticulated pythons (*Malayopython reticulatus)* in Asia. Over a five-year period, we conducted 527 riverside surveys (with mark-recapture) for this species in a protected area in Sabah, Malaysia. Detectability of pythons was dependent on moon phase but always low (mean = 20%); and the total number of pythons captured (*N* = 159 total, *N* = 83 during standardized surveys) and recaptured (*N* = 25) despite that intensive long-term effort was insufficient to enable robust mathematical modelling. Our results suggest that attempts to base sustainable-harvesting targets for *M. reticulatus* on mathematical models are futile, and that repeated surveys of the attributes of harvested specimens provide a more reliable basis from which to evaluate sustainability.

**Supplementary Information:**

The online version contains supplementary material available at 10.1038/s41598-026-57269-9.

## Introduction

Many wildlife populations worldwide are under threat from a range of factors including climate change, habitat destruction, pollution, invasive species, and pathogens (e.g.,^1,2^). For species whose bodies provide an economic benefit, over-exploitation by people for food, skins, or medicinal products may also affect the viability of wild populations^3,4^. Concerns about the sustainability of current levels of harvesting have stimulated management actions including annual quotas for offtake, harvest size limits, and in extreme cases the imposition of bans by either the countries where that resource is harvested or by countries in which that resource is processed and/or consumed^5^. In some cases, there is broad agreement about a species’ vulnerability, and consequent consensus about strict regulation of offtake. In other cases, however, there is disagreement about the impacts of harvesting levels. Some of that disagreement arises from philosophical considerations: for example, many animal welfare organisations oppose any level of commercial offtake based on ethical grounds^6^. However, other disagreements (often, between exporting and importing countries) concern the extent to which harvesting impacts population viability. To resolve such debates, we need to evaluate the sustainability of different levels of offtake.

Over a long period, sophisticated and powerful methods to evaluate sustainability of harvesting have been developed for commercial and recreational fisheries^7,8^ and for the management of “game” birds and mammals (such as waterfowl and deer) in temperate-zone Northern Hemisphere systems^9–11^. Frequently, quotas for harvest are derived from mathematical models of population viability that incorporate information on a species’ abundance and rates of growth, survival, and maturation. With a sufficiently detailed empirical base, such models can predict the numbers (and ages, sexes, etc.) of individuals that can be culled annually without reducing the species’ abundance to levels where their populations cannot recover^12,13^.

Although the population-modelling approach is mainstream for commercially harvested fishes, birds, and mammals, it has proven difficult to apply the same method to the widespread harvesting of reptiles in Asia. For example, hundreds of thousands of specimens of the world’s longest snake, the reticulated python (*Malayopython reticulatus*), are harvested from the wild in Indonesia and Malaysia each year^14–17^ for domestic and international trade in meat, skins, and body parts for medicines. The commercial-scale trade in this species has been taking place for > 100 years, suggesting that reticulated pythons continue to be abundant^15^.

Frustratingly, the biology of this species renders it difficult to quantify demographic traits in the wild. These superbly camouflaged ambush predators inhabit densely forested habitats and may remain immobile for weeks at a time; and hence avoid detection by people^18^. Biologists who have examined the carcasses of harvested snakes have concluded that the commercial offtake is sustainable, based on life-history attributes (rapid maturation, high fecundity, etc.) and long-term consistency in the numbers and attributes (sexes, body sizes, sizes at maturation, body condition, etc.) of the animals entering the commercial trade (e.g., Natusch et al.^15^, Shine^19^. Those authors argue that low detection probabilities of cryptic ectotherms in dense forests render mathematically-based population models inapplicable, because the essential field data are impossible to obtain. However, some of the countries that import python products (skins) do not accept that conclusion. For example, imports of reticulated python skins from Peninsular Malaysia into the European Union (EU) were banned in 2003 in response to a lack of information about the sustainability of harvesting^20–22^. Based on research concluding trade levels to be sustainable, the EU ban was lifted in 2017, but then reinstated in 2019 (a decision that was upheld in 2021), citing that the current trade is unsustainable^21,23^. This divergence in views centres on the role of field-based surveys and population-viability modelling for the evaluation of sustainability. Some researchers claim that the biology of the system renders such a modelling approach effectively impossible; while others contend that more effort into monitoring could provide the data needed for robust models (e.g., Nijman^24^).

To address the suggestion that more intensive monitoring efforts could yield the kinds of data required for robust demographic models, we conducted a capture-mark-recapture study of reticulated pythons in a landscape where this species might be expected to be more numerous and more easily detected than would be true across most of their range. Specifically, we worked in an area where (a) the python population is not subject to harvesting for trade, so should theoretically be at carrying capacity; and (b) we could survey preferred (riverside) habitats by boat, accessing a relatively open habitat where pythons are more obvious than in dense forest, and allowing close approach without disturbing the animals. We gathered information on encounter rates under a range of environmental conditions, to allow systematic monitoring to improve capture/encounter rates by timing surveys to coincide with peak activity levels and improve sample sizes^25,26^. Specifically, our study attempted to estimate: (i) the inter-annual and seasonal (monthly) variation in capture rates, size distributions, and body condition of reticulated pythons; (ii) detectability and occupancy of pythons within the study site, and (iii) how environmental variables affect python detectability. We discuss the results of our study in the context of finding appropriate methodologies for estimating the impacts of commercial harvesting on the long-term viability of reticulated python populations in Southeast Asia.

## Methodology

### Study site

**Fig. 1:**
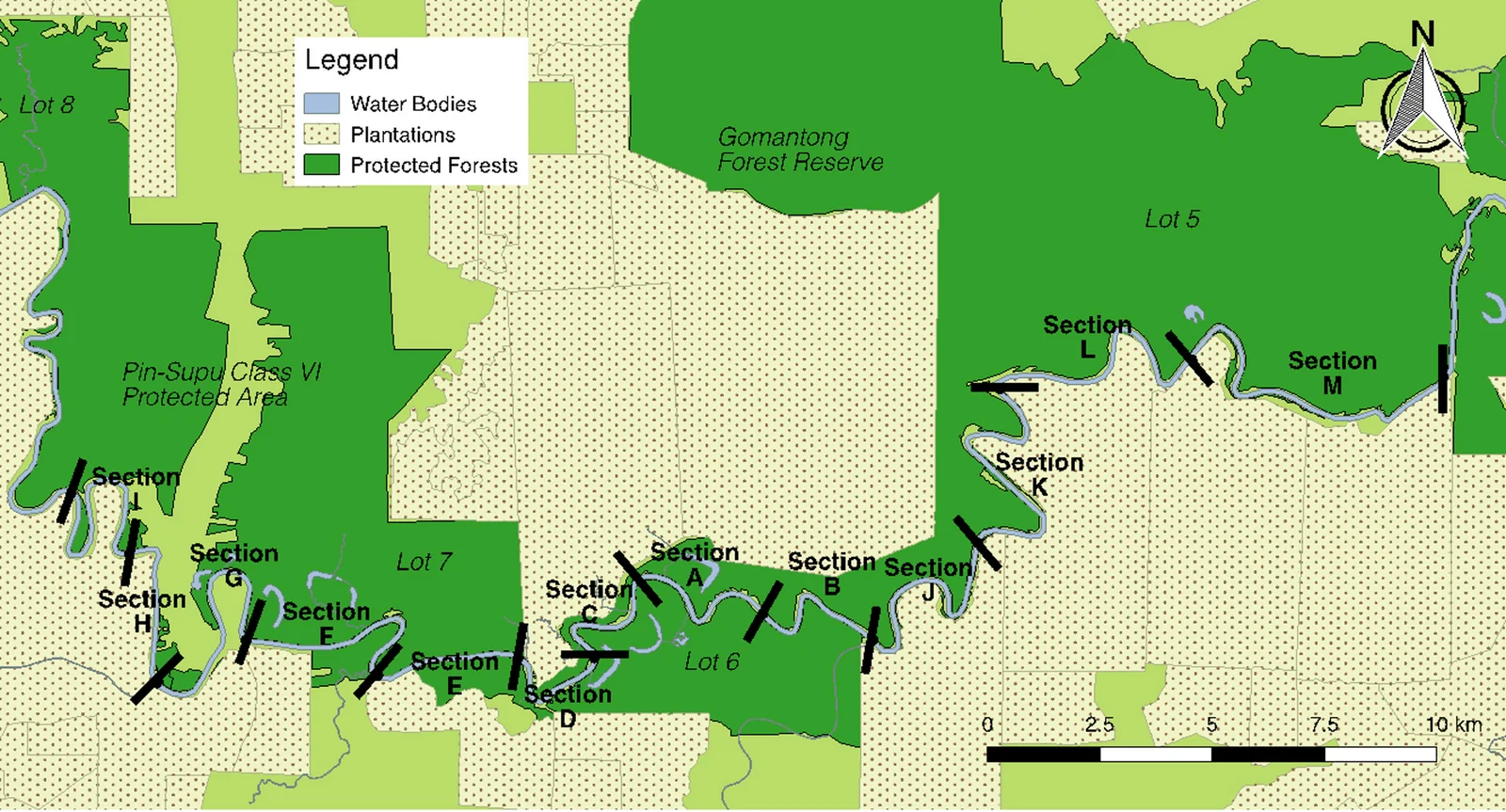
Map showing survey sections along the Kinabatangan River. Each section comprises two transects, on the North bank and South bank.

We conducted our study within the Lower Kinabatangan Wildlife Sanctuary (LKWS) and surrounding plantations and villages, in Eastern Sabah, Malaysian Borneo. The study focused on lots 5, 6, and 7 of the LKWS, and Pin Supu Forest Reserve and surrounding plantations, encompassing a 64 km stretch of the Kinabatangan River (Fig. 1). The area experiences a warm, wet, tropical climate, with year-round temperatures of 21–34˚C and annual precipitation averaging 3000 mm^27,28^. The forested habitat consists of freshwater swamp forests, semi-inundated forests, and riparian and mixed lowland dipterocarp forests designated as High Conservation Value (HCV) areas, whereas the plantations were comprised solely of oil palm. Python numbers may have been bolstered by the large-scale conversion of the region to oil palm plantations (providing abundant rodents as prey^19^: The absence of a commercial-scale meat and skin trade in Sabah allows for a baseline assessment of population demographics and abundance without the influence of trade-related mortality. Finally, the site can provide valuable insights into whether or not the pythons examined in processing facilities elsewhere in Malaysia (and in Indonesia) provide a representative sample of the wild population.

### Surveys for pythons

We undertook riverbank surveys for pythons over five years, from April 2016 to March 2021. Other studies (e.g.,^29^) have identified reticulated pythons as heavily associated with water bodies. In many areas, road transects (e.g., Hart^30^, Madsen^31^, Wilson^32^), and river transects (e.g., Lind et al.^33^, Plummer^34^) have proven to be effective methods for surveying snakes. For this reason, we used a boat to survey for pythons along the riverbank at night when the snakes are most active^35^.

We divided the 64-km stretch of river encompassing the study area into 13 approximately equal sections of 4–6 km in length, usually demarcated by obvious land features, such as tributaries, to aid navigation. We considered the northern and southern riverbanks within each section to be separate transects, resulting in 26 transects totalling 128 km in length. One section (therefore two transects – the North and South banks) was surveyed each night, between 1900 and 2200 h. On average, we undertook 18 transect surveys per month, with variations in that number dependent on logistical constraints and other fieldwork (range: 0–34 survey transects per month, or 0–17 survey nights per month). If pythons were captured, they were returned to their capture location the following night. A fully randomised survey design was not used for logistical reasons (including fuel economy).

We conducted river surveys with a minimum of three people at a low speed (4–6 km/h) and close to the riverbank (2–10 m, depending on water depth). We used strong head torches (e.g., Ledlenser H14R.2–1000 lumens) and spotlights to search for pythons among the undergrowth or on the bare riverbank. We did not undertake surveys in dangerous weather (heavy rain and thunderstorms), or when the river was in flood. During long periods of drought, we did not undertake surveys because overgrown vegetation rendered snakes difficult to see. Due to logistical constraints, we did not survey all transects an equal number of times.

Our standardized river surveys were augmented by opportunistic capturing of any pythons that we located in the study area. This included while travelling between survey sites along the riverbank, and while walking along trails in forested areas or adjacent oil palm plantations. We were also brought several pythons by local people.

### Capture and measurement protocol

We recorded the location of each python encountered using a handheld GPS unit (Garmin GPSmap 64 s). All pythons were collected (if possible), placed in cloth bags, and transported back to the Danau Girang Field Centre (Fig. 1). The next morning, we probed each specimen to determine its sex and measured snout-to-vent length by stretching the snake alongside a steel tape measure. A passive integrated transponder (PIT) tag was inserted under the skin to identify individuals during subsequent recaptures. We released pythons at their capture location within 24 h of capture.

### Environmental variables

Each evening when transect surveys were undertaken (2000 h) we recorded environmental variables at Danau Girang Field Centre. We measured temperature (to the nearest 0.5˚C) and relative humidity (to the nearest 0.5%) using an easylog datalogger (Lascar electronics), and rainfall to the nearest 0.5 mm using a rain gauge. We determined the moon phase at the time of the survey using Moon Phase Calculator (Probadosoft) as percentage of the lunar surface illuminated at the study location. Because lux meters with sufficient sensitivity to calculate true luminosity during each survey were not available, and the moon may have been obscured by trees or clouds, we used moon phase as a continuous variable to calculate the stage of the lunar cycle, rather than lunar brightness per se.

### Data analysis

At the outset of this research, part of our goal was to estimate reticulated python population sizes and vital rates at our study site using traditional mark-recapture analysis methodologies (e.g., an open-population Jolly-Seber model^36^. However, the low number of recaptures during systematic (i.e., comparable) surveys precluded this method. Our analyses below present other methods used to examine encounter rates and population dynamics of pythons at our study site. We conducted all analyses in R (version 3.5.0, R development core team 2020).

#### Encounter/capture probabilities, sexes and body sizes

Because our survey transects were unequal in length, we calculated encounter rates as the number of pythons recorded for every kilometre surveyed. We used chi-squared tests to examine differences between encounter rates between the north and south banks of the river.

To assess capture probabilities across months, we used a General Additive Model (GAM) with log link and Poisson errors using the package *mgcv*^37^. We modelled month as a cyclic cubic regression spline, with year and number of surveys per month as covariates. We performed model comparisons using AIC and used sex as either an interaction term (monthly splines were allowed to vary between sexes) or as a covariate to assess whether monthly capture rates differed between males and females. Individuals that were encountered and escaped capture were excluded from this analysis, as their sex was unknown.

We examined differences in the mean body sizes of captured pythons using a generalised linear mixed model (GLMM) with year as an explanatory variable, sex as a covariate, and month as a random effect using the package *lme4*^38^. For all the models assessing trends, *post hoc* contrasts of marginal means (for categorical variables) or marginal trends (for where slopes were allowed to vary between categories) were carried out using the package *emmeans*^39^, using the Tukey adjustment for p-values. Individuals that were encountered and escaped capture were excluded from this analysis, as their sex and size was unknown.

We used a binomial GLMM to explore the effects of environmental variables on the probability of encountering pythons along riverbanks. We used a binomial outcome variable (whether or not at least one python was encountered along a surveyed transect) to create a global model with total rainfall that day (mm), rainfall the previous day (mm), moon phase (as the percentage of illumination of the moon surface), temperature (˚C) and relative humidity (%) as explanatory fixed effects. We included the transect surveyed as a random effect to account for differences in encounter probabilities between river sections. All possible fixed effect combinations were examined using the package *MuMIn*^40^ and ranked using AICc. We considered all candidate models within < 2 AICc to be equivalent.

For all models examined, continuous variables were checked using the package *olsrr*^41^ for collinearity, and models were examined using the package *DHARMa*^42^ to perform residual diagnostics. Likelihood ratio tests for significance of fixed effects were performed by fitting equivalent models with and without the fixed effect of interest, using the package *lmtest*^43^. Model predictions were calculated using the package *ggeffects*^44^ and data were plotted using *ggplot2*^45^. For mixed-effects models, marginal and conditional R^2^ values were calculated with the package MuMIn^40^.

#### Occupancy and detectability

Occupancy modelling is an effective technique for monitoring species that are difficult to detect, or where detectability is variable^46,47^. In contrast to the mixed model method used above, occupancy models can use site-level covariates to estimate whether transects where no captures occurred are likely to be real absences or a result of the difficulty of detection.

We undertook occupancy modelling using the R package *unmarked*^48^. Observation-level categorical variables included month and year whereas moon phase, temperature, humidity, total rainfall that day, and rainfall the previous day were included as covariates. Site-level categorical variables included riverbank (north or south), with transect length and proportion of agricultural land within buffer zones of both 250 and 500 m from river (calculated within QGIS) included as covariates. We first examined a global model with all covariates, and then all possible covariate combinations using the dredge function in the package MuMIn^40^. We ranked all models using AIC corrected for sample size. We report all models within <2AICc of the top performing model, as well as the starting and null models. The top-performing model was checked for goodness-of-fit using a bootstrapped Mackenzie-Bailey test, with 500 simulations, using the package *AICcmodavg*^49^.

## Results

### Python encounters

A total of 527 systematic riverbank surveys were carried out over five years, resulting in 2,427 km of surveys. During these surveys, a total of 83 reticulated pythons were located. Pythons were also captured opportunistically in the study areas outside of dedicated surveys, resulting in an additional 76 pythons captured (total = 159; 75 females, 74 males, and 10 unassigned hatchlings). Of this total, 25 individuals were recaptured on at least one occasion, either during surveys or opportunistically, across 40 total recapture events. One further marked individual was ‘recaptured’ after finding its carcass in the forest. There were 10 occasions where a python that was visually located escaped capture, giving a 95.2% capture success rate overall (of 209 total capture attempts). There were also four occasions where captured pythons escaped their enclosures at the field centre before measurements could be taken.

### Spatial differences in encounter rates

**Fig. 2:**
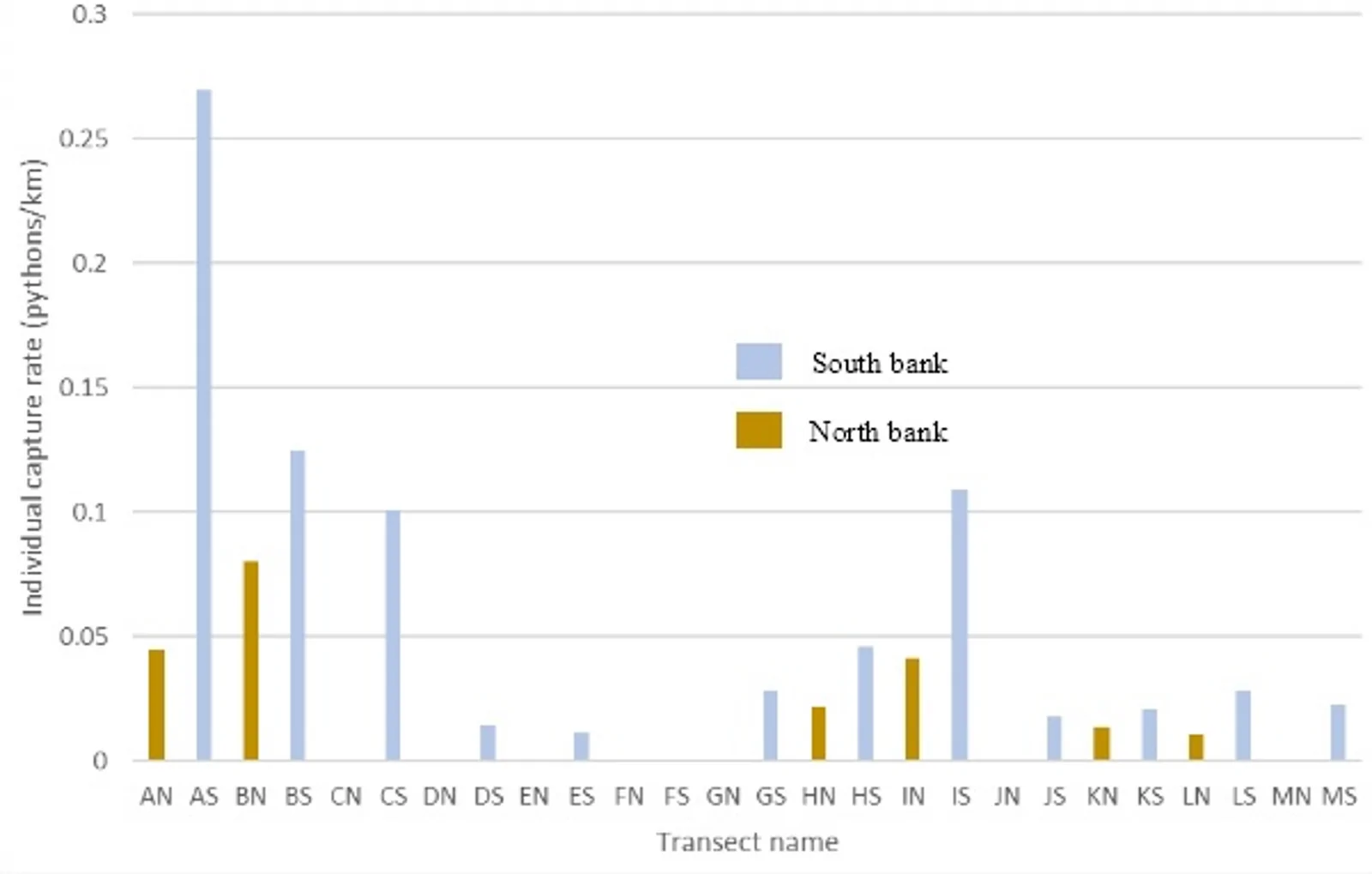
Capture rate of individual pythons (i.e., excluding recaptures and uncaught encounters) for each transect, controlled for differing transect distance and number of surveys -. N = North bank, S = South bank, for each transect section (A-M). Transects in sections G-M were not surveyed during the first year of data collection.

Seventy-nine python encounters (including recapture events) occurred on the southern bank of the Kinabatangan River, while fewer (18) specimens were encountered on the northern bank (χ^2^ = 34.37, p = < 0.001). However, when split by transects the difference was found not to be significant (χ^2^ = 8.68, *p* = 0.67), because of atypically high encounter rates along one transect on the southern bank. Pythons were found in 12 of 13 transects on the southern bank, but in only 6 of 13 transects along the northern bank (Fig. 2), despite equal survey effort on both banks.

### Annual and seasonal trends in encounter rates

**Fig. 3:**
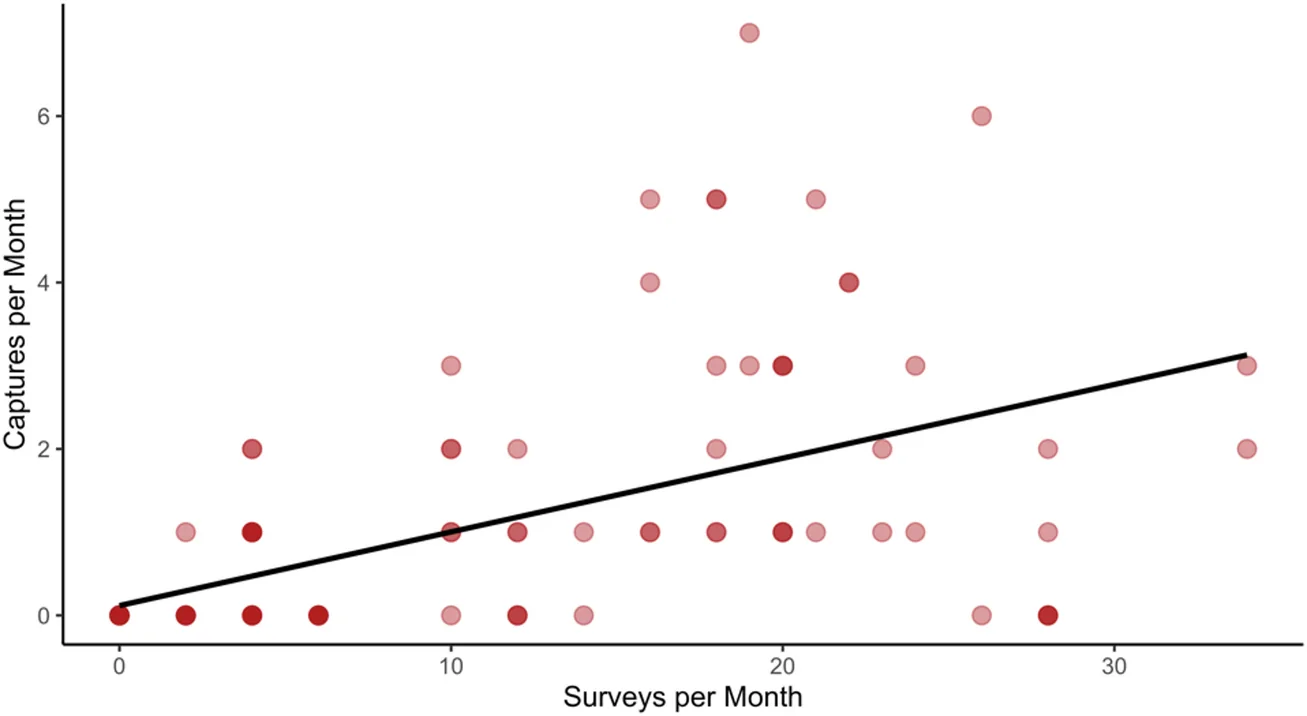
The relationship between the number of transect surveys carried out per month and the number of pythons captured per month. Darker coloured dots represent overlapping points.

Due to logistical constraints related to weather and river levels, the number of surveys carried out in different months was unequal (ranging from 0 to 34). A linear regression did not meet assumptions of normality of residuals, and Spearman’s rank showed a significant monotonic relationship between the number of surveys and number of captures per month (S = 96960, rho = 0.663, p = < 0.0001) (Fig. 3). This potential bias was incorporated into our assessment of the number of pythons caught over time.

**Fig. 4:**
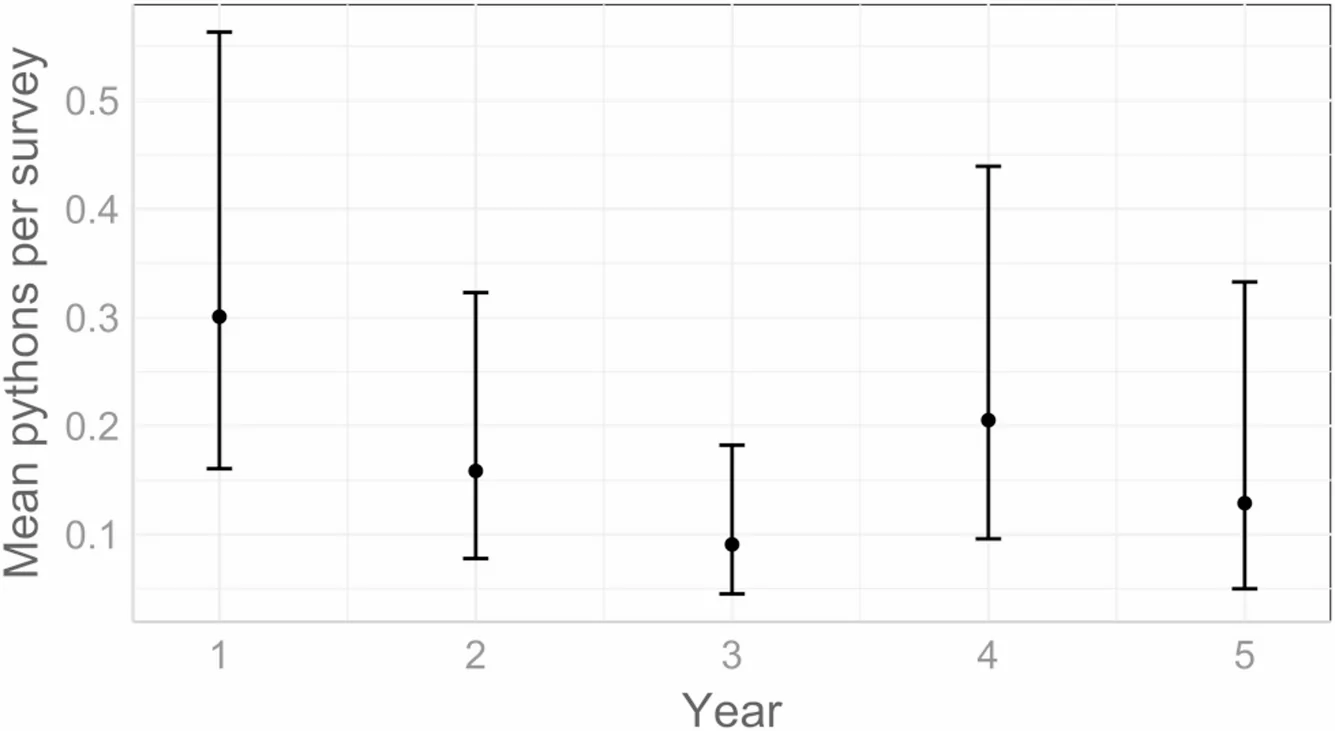
Mean capture rate in each year from a GAM. Year 3 had a significantly lower capture rate than year 1. Capture rate is given as the number of pythons caught per survey, by controlling for number of surveys and setting the value for model predictions at 1. Data for males and females are pooled.

Our GAMs assessing trends in encounter rates over time suggest that there were not enough data to effectively detect differences between the sexes, which were not significant (t = 0.095, *p* = 0.925). Encounter rates by year (controlling for differences in monthly capture rates) were only significantly different between years 1 and 3 (Fig. 4; supplementary Table 2.1).

**Fig. 5:**
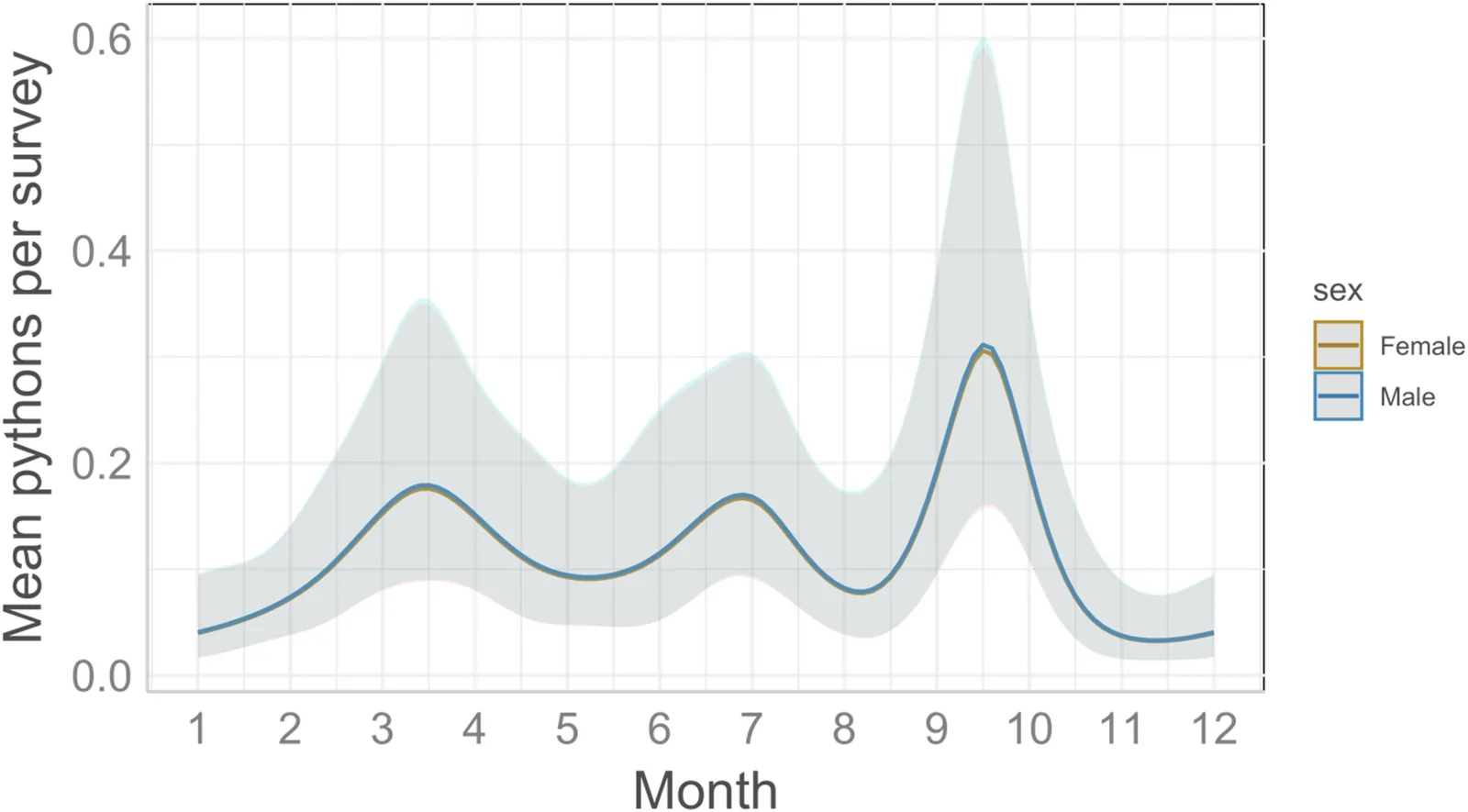
The trend in mean capture rate across months for male and female pythons, based on predictions from a General Additive Model. Capture rate is given as the number of pythons caught per survey, by controlling for number of surveys and setting the value for model predictions at 1. Capture rates for males versus females did not differ and therefore overlap completely.

Monthly variation in capture rates showed that model spline terms were highly non-linear (edf = 6.39), with a peak around September and October, and two smaller peaks around March/April and July (Fig. 5). The model overall showed moderate fit, with 53.6% of deviance explained.

### Demography of encountered pythons

A full set of morphological measurements was obtained from 146 individuals (74 females and 72 males). Mean snout-vent length (SVL) was 226.7 cm (± SD 85.4 cm) whereas mean mass was 5.74 kg (± SD 4.89 kg). Females were longer than males, with female SVL averaging 236.3 cm (± SD 92.9) and males averaging 216.9 cm (± SD 76.2).

**Fig. 6:**
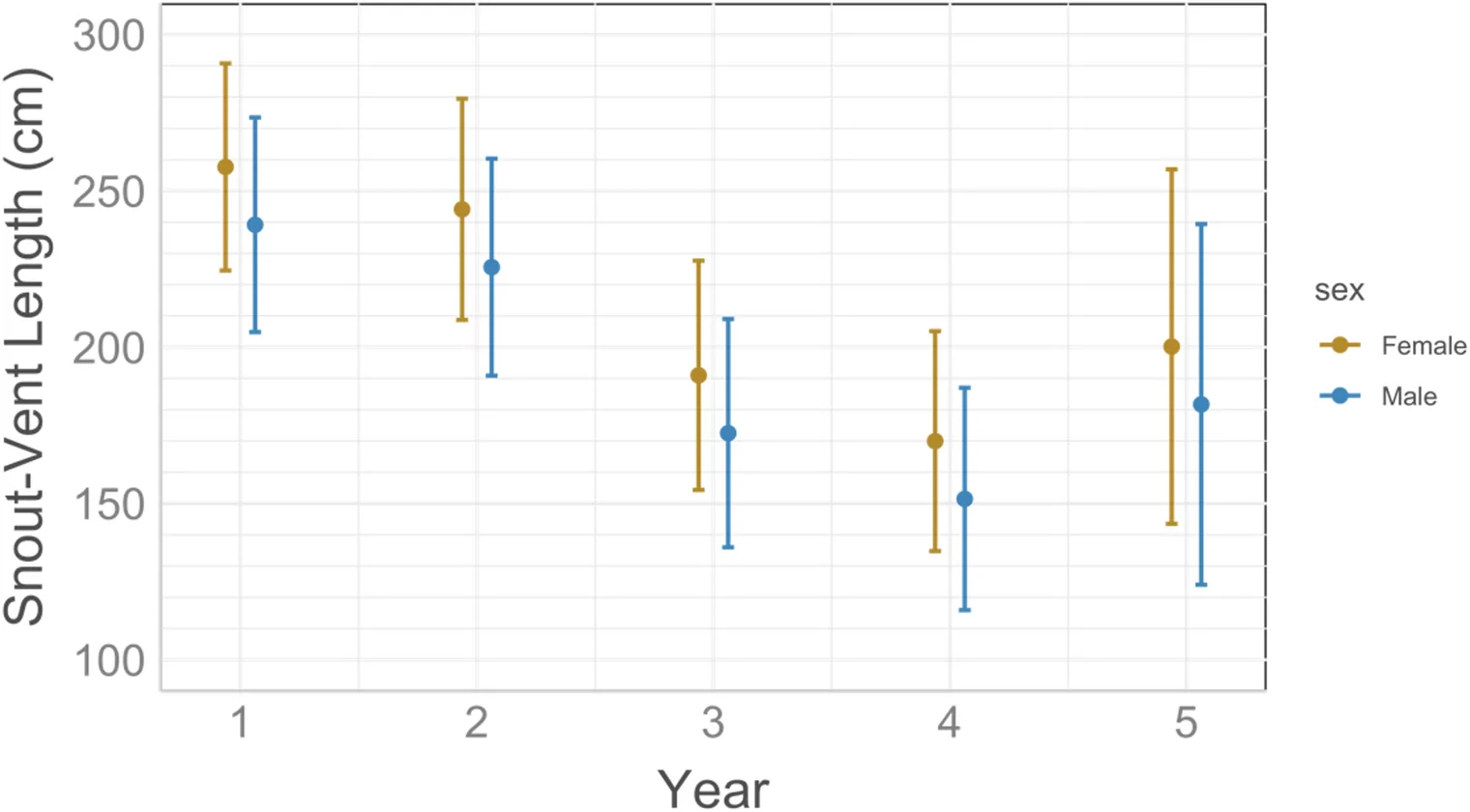
Predicted values from a GLMM examining variation in the mean body sizes (as snout-vent length) of pythons caught across years.

Our overall GLMM showed that the mean body sizes of captured pythons varied among years (χ^2^ = 54.50, p = < 0.0001; Fig. 6; see supplementary Table 2.2). Females were larger on average than males in most years (χ^2^ = 8.95, *p* = 0.003; Fig. 6), but the lack of statistical significance of some large disparities in mean SVL among years (up to 57.5 cm) suggests poor statistical power for detecting trends – most likely arising from small sample sizes and high variance (supplementary Table 2.2). The marginal R^2^ of the model was 0.206, whereas the conditional R^2^ was 0.327.

### Environmental predictors of encounter rate

**Fig. 7:**
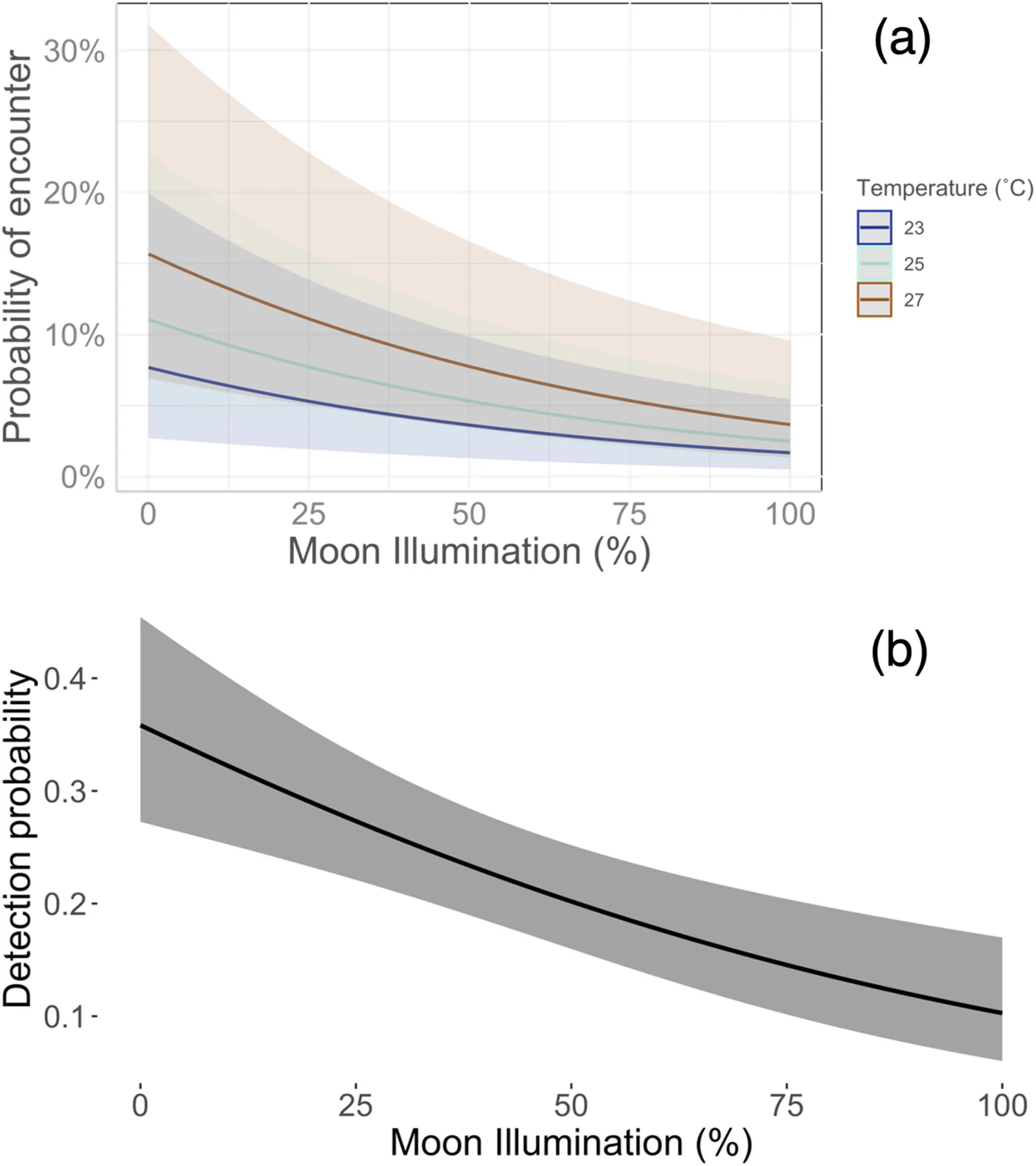
(**a**) Predictions of how moon illumination affects the probability of encountering at least one python during any given survey, whilst statistically controlling for the differing encounter rates in different transects, using a binomial mixed model, and (**b**) model predictions from the chosen single-season occupancy model showing the effect of moon illumination on detection probability.

Examination of potential models revealed five candidate models to be within 2 AIC of each other (Supplementary Table 2.3). The best model had moon and temperature as fixed effects and was chosen based on the most parsimonious model having non-significant quantile deviations when model checks were performed. Only the moon illumination percentage was found to correlate significantly with encounter probability in a likelihood ratio test (Supplementary Table 2.4); encounter probability decreased with increasing moon illumination (Fig. 7a). Although not statistically significant, there was a trend for higher encounter rates at higher temperatures (Fig. 7a).

### Occupancy modelling and detectability

The overall null mean site occupancy across all transects (i.e., with no covariates included) was estimated to be 0.662 ± 0.094, while null detection probability was 0.221 ± 0.021. With the addition of environmental variables, three candidate models were within <2AICc of each other (Supplementary Table 2.5), but only the top model showed good fit after performing a Mackenzie-Bailey goodness of fit test (c-hat = 1.45, *p* = 0.18). The chosen model had a mean occupancy probability across all transects higher than the null model at 0.718 ± 0.136, while the mean detection probability was lower than the null model at 0.205 ± 0.024. Occupancy rates were significantly higher on the south compared to the north bank of the river (93 vs. 32%: z = 2.54, *p* = 0.0112; Fig. 7b). Although transect type (forest or corridor) was included in one model, this was not statistically significant (z = 0.71, *p* = 0.478). Detection probability decreased with moon illumination (z = −3.80, *p* = 0.0001), with a full moon having only 28.6% of the detection probability of a new moon (Fig. 7b). Humidity was included in one candidate model, but was not significant (z = −0.99, *p* = 0.323). Month, year, temperature, and daily rainfall on the day of the survey and the previous day did not appear to correlate with detection probability.

## Discussion

Our five-year study is by far the most intensive research project ever conducted on free-ranging reticulated pythons. Our results provide a stark example of how difficult it is to find this giant species in their natural habitat. Although we worked in a protected area where pythons were not disturbed or removed by hunters, and used a method (nocturnal spotlight surveys from a boat) that gave us a clear view of preferred habitats in a way that was unlikely to alert snakes to our approach, we only found 83 pythons in 2,427 km of riverbank in the course of 527 systematic surveys. Our analyses show low detectability as a major problem: we rarely saw pythons even in areas where they were known to exist. Given the generalist habitat use of this species, and the superficially uniform habitat over the entire survey area, it is almost certain that reticulated pythons occurred in all the areas that we surveyed. Nonetheless, we failed to detect even a single python in 25% of those sites.

This result will not be surprising to field biologists with experience in the tropics. Almost 30 years ago, Shine et al.^19^, wrote (p. 62–63) *“it is virtually impossible to obtain reliable information on population densities of large snakes inhabiting complex tropical habitats*,* at least on a spatial scale large enough to permit generalizing to broader areas. The problems are several. First*,* most pythons are well-camouflaged ambush predators*,* lying in wait for passing prey items. The snakes are hence immobile for long periods and are virtually impossible to find at these times. Second*,* the tropical climate removes any need for the snakes to bask to raise their body temperatures*,* so that another common reason for snakes to move about (and thus*,* become available for capture) is removed”.* Nothing that has been learned about the ecology of tropical snakes since 1998 – and there has been substantial research – has modified those conclusions. Below, we review this literature in the context of applying Population Viability Analysis (PVA) to assess sustainable offtake levels for reticulated pythons.

To construct PVA models that accurately predict population-level responses at a broad spatial scale and over meaningful time periods, we need robust estimates of several parameters including abundance, birth rates, death rates, age structure, age at maturation, and rates of immigration and emigration (e.g.,^50^). Is it feasible to obtain such data for our study system? Reviews of research on the critical variables needed for a PVA model have concluded that such data are well-nigh impossible to gather for snakes in general because of their low detectability and frequent inactivity^51,52^. That low detectability violates important statistical assumptions required for the use of population models^53^. Exceptions may include situations where snakes aggregate in response to a scarcity of some critical resource, such as overwintering sites^54^. Reticulated pythons are not known to aggregate.

How general is low detectability for tropical snakes? Detectability of the Burmese python (*Python bivittatus*) has attracted research because this species is a damaging invader of North American ecosystems. One enclosure experiment reported that from a possible 190 detections, only 2 occurred (1%; Dorcas & Wilson^55^). Another study estimated Burmese python detectability probability to be between 0.0001 and 0.0146 using a combination of transect surveys and known locations of telemetered pythons in the vicinity of transects^56^. For reticulated pythons, surveys using passive live-baited traps and nets yielded low catch-per-unit-effort and overall sample sizes^21,29^. Disappointing results also have emerged from alternative field-based survey approaches such as passive capture via drift fences and traps, and the use of sniffer dogs^57^. A separate study conducted in Sabah on snake detectability found that reticulated python encounters were too low to assess, with 5 encounters from 295 walked transect surveys^58^. We have seen no evidence that existing field methods can provide robust estimates of population-level traits in a species such as the reticulated python.

For reliable prediction of harvest impacts, the PVA also must be validated from multiple populations over time periods that capture realistic levels of spatiotemporal variation. Thus, data from a short-term study at a single site (even if it was achievable) would not be sufficient for overall conclusions about harvest sustainability over larger spatial scales and longer time periods. That weakness is less of an issue if the taxon in question exhibits highly conservative demographic traits (abundances, birth and death rates etc.) across broad areas and over long timespans.

Mark-recapture studies on tropical snakes enable us to evaluate the magnitude of spatiotemporal variation in demography. Most studies of this kind have been short-term or have relied on small sample sizes (e.g.,^59–61^) but a few studies have looked over longer periods with larger sample sizes. All those latter studies have documented enormous variation in vital rates and abundances through time. For example, the abundance, growth rates, body condition, reproductive output and survival rates of water pythons (*Liasis fuscus*), keelbacks (*Tropidonophis mairii*), slatey-grey snakes (*Stegonotus australis*) and file snakes (*Acrochordus arafurae*) at a site in tropical Australia varied strongly among years depending on rainfall-driven fluctuations in prey abundance (e.g., Brown and Shine^62^, Brown^63^, Madsen^64^, Madsen^65^, Madsen^31^). The magnitude of impacts was strong enough to prevent populations from ever attaining stable age/size distributions^66^. Occasional extreme rainfall conditions (floods or droughts) virtually extirpated populations in some cases, with impacts falling in complex ways according to an individual’s sex and body size^67,68^.

Longterm mark-recapture studies on snakes also have revealed strong spatial divergences in demography. For logistical reasons, most ecological research on tropical snakes has relied upon single study sites; but in three species of sea snakes from New Caledonia (*Emydocephalus annulatus* by^69^; *Laticauda laticaudata* and *L. saintgironsi* by^70,71^, local populations (even closely adjacent ones) showed asynchrony in annual fluctuations in demographic traits; and strong overall divergences in parameters such as age structure.

Although field data thus are insufficient to support robust population modelling, fieldwork is valuable in revealing factors that drive activity and, hence, catchability. In the present study, we found more pythons on dark (new-moon) nights than on lighter (full-moon) nights. Similar effects of moon phase on capture rates of snakes have been reported in a variety of species in a range of continents^72–76^. Also consistent with our own results, analyses of capture rates of invasive Burmese pythons showed higher catch per unit effort in warmer conditions^77^. Studies on abundant watersnakes indicate that capture rates are influenced by a multitude of external (e.g., weather and season-related) and intrinsic (e.g., sex, reproductive state) factors (e.g., Willson et al.^78^).

Even within a single sympatric snake assemblage, species may vary considerably both in their detectability and in the factors that drive variation in that detectability^47^. Small shifts in microhabitat use can massively affect snake detectability^79^. In a tropical rainforest area close to our own study site in Borneo, detectability of riparian snakes shifted strongly among years and seasons and was affected by prior rainfall^58^. In short, studies on detectability of snakes in natural environments reveal huge biases associated with a multitude of factors. That sensitivity confounds any attempt to infer underlying abundances from encounter rates; and low rates of recaptures preclude any meaningful estimation of rates of growth, reproduction, or mortality.

How, then, can we evaluate sustainability of the commercial trade in reticulated pythons? The consensus from published literature (and our own research) is that logistical problems make it effectively impossible to obtain the ecological data required to construct reliable population-viability models for such a species in such a habitat. For example, large disparities in capture rates and mean body sizes over time in our own results are almost certainly due to low sample sizes generating variation rather than to changing population densities or body size distributions. Attempts to model harvest impacts or sustainability from such data thus would be futile. Population-viability analyses are useful for predicting sustainable levels of offtake, but they are not a panacea. There are important situations in which they are simply not feasible. The case of large pythons in tropical rainforests is one example of such a situation.

Fortunately, an alternative approach to assessing sustainability is available. Dissection of harvested animals at processing facilities can provide massive datasets that not only clarify fundamental aspects of the species’ ecology (diets, reproductive rates, etc.; all relevant to a species’ resilience to harvesting) but also allow for straightforward comparisons of offtake (numbers, sizes, sexes, maturation levels, condition, etc.) through time^14–17,21,22,29,80^. Stability in those parameters is indicative of a population’s ability to persist despite commercial harvesting^19,22^.

We note, however, that any method entails biases. For example, juveniles are underrepresented in the commercially-harvested samples because small individuals do not have commercial value; very large individuals are also more likely to be scarred and have less value for their skin; and gravid females may sometimes be left by hunters to ensure population sustainability^15,21,81^. Also, because many pythons are sourced from local people and because middlemen are involved in trade, it can be difficult to obtain accurate information about the locations from where animals were captured^14,15,21^.

In summary, our field results support the suggestion advanced many years ago by Shine et al.^19^: that analyses of culled reticulated pythons offer a straightforward way to evaluate harvest sustainability, whereas the superficially more sophisticated approach of field surveys and mathematical population modelling does not. A simulation model is only as good as the data used to develop and test it; and for ambush-foraging snakes in rainforests, even herculean field efforts such as our current study fail to generate enough reliable information to construct such a model. In the absence of a model, we can decide either to rely upon information gathered from culled animals or to significantly restrict the trade because we can never obtain the kinds of information needed to build the kinds of models that are suitable for more-easily-monitored wildlife.

## Supplementary Information

Below is the link to the electronic supplementary material.

Supplementary Material 1

## Data Availability

Data are available from the authors upon reasonable request.
